# Characterizing particle size in agricultural runoff to inform stormwater basin design

**DOI:** 10.1002/jeq2.70267

**Published:** 2026-09-21

**Authors:** Michael A. Holly, Andrew J. Votis, Mason Anderson‐Giles, Kpoti M. Gunn

**Affiliations:** ^1^ Resch School of Engineering University of Wisconsin‐Green Bay Green Bay Wisconsin USA

## Abstract

Upper US Midwest sediment‐basin design commonly relies on parent‐soil texture classes and texture‐based settling‐velocity defaults (Natural Resources Conservation Service Conservation Practice Standard Code 350), yet conservation‐managed fields can export fine particles that bypass gravity settling. Runoff from 13 edge‐of‐field watersheds in Wisconsin and Indiana showed systematic divergence between particle size distribution representations: volume‐weighted (mass‐based) D_50_ ≈ 30 µm (coarser classification) versus number‐weighted (count‐based) D_50_ ≈ 3.5 µm (clay‐rich suspension). A mixed‐effects framework identified seasonality and water chemistry as stronger predictors than agronomy, including winter coarsening (*β* ≈ +4.35, *p* < 0.001), consistent with low‐density organic flocs inflating apparent size without proportional settling benefit. Nutrient partitioning diverged by fraction, with total phosphorus yield decreasing with D_50_ (*p* < 0.001) while total nitrogen yield increased with D_50_ (*p* = 0.014), supporting phosphorus coupling to fine mineral particles and nitrogen association with larger organic aggregates. Fine‐particle dominance limits gravity settling in sedimentation basins, so substantial reductions in suspended sediment and turbidity for agricultural runoff typically require coagulation/flocculation in addition to hydraulic detention.

## Core Ideas

- Mass‐ and count‐based particle size distributions classify the same agricultural runoff as coarse versus clay‐rich suspensions.
- Conservation management can shift exported sediment toward fine particles that bypass basin settling.
- Winter events can inflate apparent particle size through likely low‐density organic flocs without proportional settling benefit.
- Nutrient export partitions by fraction, with total phosphorus tracking fine mineral particles and total nitrogen associating them with likely larger organic aggregates.
- Coagulation/flocculation is required for substantial suspended sediment and turbidity reduction when fine particles dominate.

## Plain Language Summary

Sediment basins slow runoff so soil particles can settle before water leaves a field. This study measured particle sizes in runoff from 13 agricultural sites in Wisconsin and Indiana. Results showed that runoff can appear coarse when measured by mass, but count‐based measurements showed many small particles that are harder to remove by settling. These fine particles can carry nutrients and may pass through sediment basins. Measuring runoff particle size can help design agricultural treatment systems that better match the particles carried from working fields.

**Abbreviations**
ANOVA: analysis of variance
BMP: best management practice
DP: dissolved phosphorus
LMM: linear mixed‐effects model
NRCS: Natural Resources Conservation Service
PAM: polyacrylamide
PSD: particle size distribution
TN: total nitrogen
TP: total phosphorus
TSS: total suspended solids

## 1 INTRODUCTION

Deployment of best management practices (BMPs) to improve soil health has inadvertently created a hydraulic challenge to unmitigated transported sediment. In‐field practices such as cover crops, no‐till, and rotational grazing are highly effective at load reduction with significant reductions in sediment loads, total phosphorus (TP), and total nitrogen (TN) (50%–90%, 30%–60%, and 40%–75%, respectively) (Blanco‐Canqui & Ruis, 2018; Haan et al., 2006; Triplett G. B. & Dick, 2008). However, these practices fundamentally alter the sediment composition reaching the field edge (Williams et al., 2009; Young et al., 2023). The preferential retention of coarse particulates near the surface creates an enrichment effect that fractionates runoff into a suspension dominated by fine‐grained mineral fractions; although these fines typically comprise only ∼20%–30% of the total eroded mass, they can transport ∼75%–90% of the associated nutrient load (Blanco‐Canqui & Ruis, 2018; Sharpley et al., 1992; Vadas et al., 2015).

The colloidal behavior of remaining suspended sediment fines drives a coupled impairment mechanism. Physically, their high specific surface area efficiently scatters photosynthetically active radiation, suppressing the benthic oxygen production necessary for aquatic resilience (Davies‐Colley et al., 1993). Chemically, this same surface area maximizes sorption capacity for phosphorus, delivering a bioavailable nutrient load that fuels algal proliferation and subsequent biological oxygen demand (Correll, 1998). By physically limiting oxygen production while chemically fueling oxygen consumption, fine‐grained sediment loads accelerate the formation of hypoxic zones (Chretien et al., 2016; Sharpley et al., 1992; Verbree et al., 2010). Establishment of this fining sequence creates an engineering paradox, that is, BMPs designed to protect soil health produce a runoff profile that is more chemically active and inherently difficult to treat via traditional sedimentation (Ghadiri & Rose, 1991).

Structural BMPs, including stormwater ponds and sediment basins, are a final opportunity for mitigation of transported suspended sediment and nutrients. Efficacy of such structural measures remains fundamentally dependent on engineering design, which is largely governed by the particle size distribution (PSD) of the incoming influent constituent profile. Fundamental physics, governed by Stokes' Law, dictates that settling velocity is proportional to the square of the particle diameter. For idealized laminar settling of spherical particles, settling velocity can be estimated as:

$$\;V_{s}=\frac{g\left(\rho_{p}-p_{w}\right)d^{2}}{18\mu }$$

where $V_{s}$ is settling velocity, *g* is gravitational acceleration, $\rho_{p}$ is particle density, $p_{w}$ is water density, d is particle diameter, and μ is dynamic viscosity. Consequently, a shift in the dominant particle size from a medium silt (30 µm) to fine silt (4 µm) increases the required hydraulic residence time 50‐fold (Verstraeten & Poesen, 2000). Urban stormwater studies provide useful sedimentation benchmarks, with suspended sediment reductions of up to 94%, but performance remains highly dependent on particle size (Clary et al., 2020). Agricultural runoff may exhibit different PSDs because runoff from working fields can contain fine mineral particles and low‐density organic‐mineral flocs. When fine particles dominate, sedimentation can be more effective as part of a treatment train that combines hydraulic detention with surface withdrawal, flow baffling, or coagulant‐assisted flocculation to improve capture of persistent suspended solids (Bhardwaj & McLaughlin, 2008; Fang et al., 2015; Kang et al., 2018; Millen et al., 1997). In pond–wetland systems treating agricultural runoff, where suspended solids removal is achieved through sedimentation, the dominance of very fine influent particles (D_50_ ≈ 2.8–4 µm) can directly limit particulate removal, consistent with the observed range of suspended‐sediment load reductions (44%–72%) and particulate‐P reductions (22%–59%) (Holly et al., 2026). Particle size characterization is therefore critical for understanding removal processes and informing system design.

**Core Ideas**

- Mass‐ and count‐based particle size distributions classify the same agricultural runoff as coarse versus clay‐rich suspensions.
- Conservation management can shift exported sediment toward fine particles that bypass basin settling.
- Winter events can inflate apparent particle size through likely low‐density organic flocs without proportional settling benefit.
- Nutrient export partitions by fraction, with total phosphorus tracking fine mineral particles and total nitrogen associating them with likely larger organic aggregates.
- Coagulation/flocculation is required for substantial suspended sediment and turbidity reduction when fine particles dominate.

Sediment basin design guidance for capturing and detaining sediment‐laden runoff is provided through the Natural Resources Conservation Service (NRCS) Conservation Practice Standard Sediment Basin (CPS 350). The national standard applies to urban land, construction sites, agricultural land, and other disturbed lands, and state‐adopted versions are published through the NRCS Field Office Technical Guide (USDA‐NRCS, 2026). In Wisconsin, the NRCS Sediment Basin standard (USDA‐NRCS‐WI, 2016) sizes the basin treatment surface area from the 1 year, 24‑h principal‑outlet peak outflow using a soil texture‐based settling velocity (*v_s_*) in *S_a_* = 1.2(q_out_/v_s_), prescribing *v_s_* = 0.0012 ft s^−^^1^ (sandy, *d* = 20 µm), 0.000073 ft s^−^^1^ (loamy/silty, *d* = 5 µm), and 0.000012 ft s^−^^1^ (clayey, *d* = 2 µm). A practical limitation is that such tabulated velocities are typically consistent with Stokes‑type formulations parameterized with mineral grain specific gravity ≈2.65 (quartz), which may not represent the porous, flocculated fines common in agricultural runoff (Droppo et al., 1998; Nghiem et al., 2024); PSD characterization for agricultural runoff could improve design standards. Measured agricultural runoff PSDs are therefore needed to evaluate whether parent‐soil‐texture‐based settling assumptions adequately represent the particles transported from working fields. PSD characterization across 13 edge‐of‐field monitoring sites in Wisconsin and Indiana, measured in the current study, provides the empirical data needed to evaluate how agricultural runoff differs from design assumptions commonly used for sedimentation‐based treatment. The objectives of this study were to (1) characterize mass‐ and count‐based PSD variability across agricultural runoff sites with differing parent soils, management practices, and seasons; and (2) compare measured agricultural runoff PSDs with texture‐based sedimentation basin standards used to inform sedimentation basin sizing. Results were then used to inform agricultural runoff treatment design considerations, including the potential contribution of sedimentation to overall treatment performance.

## 2 MATERIALS AND METHODS

### 2.1 Site locations and characteristics

**FIGURE 1:**
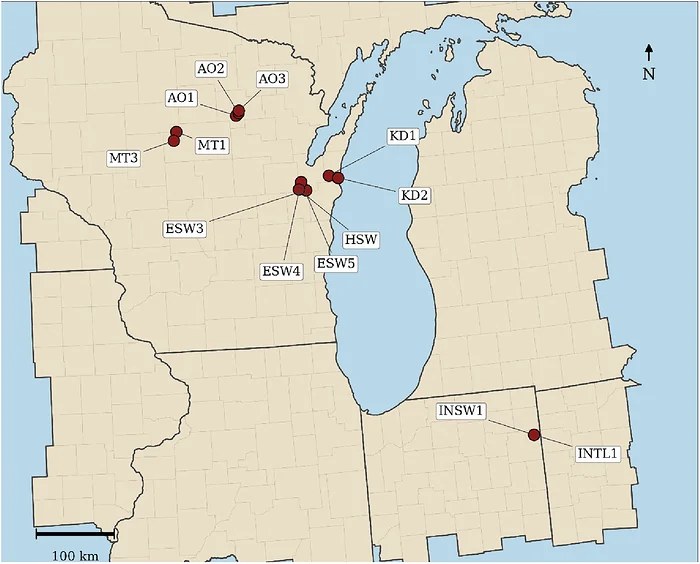
Locations of the 13 edge‐of‐field agricultural runoff monitoring sites in Wisconsin and Indiana.

Characterization of agricultural PSD utilized runoff samples from a network of 13 edge‐of‐field monitoring sites distributed across Wisconsin and Indiana. Geographic distribution of the network encompassed Langlade (3), Kewaunee (2), Marathon (2), Outagamie (1), and Brown Counties (3) in Wisconsin, alongside comparative sites in Allen County (2), Indiana (Figure 1). Information on crop management (crop type, tillage operations, and fertilizer applications) was collected through direct consultation with the farmer (Table 1). Soil type at each sampling location (Table 1) was identified using the US Department of Agriculture Web Soil Survey (Soil Survey Staff, 2025).

**TABLE 1:** Summary of monitoring site locations and characteristics.

Site ID	Location	Parent soil classification (WSS)	Watershed area (ha)	Primary cropping system	Dominant tillage practice	Sample size
AO1	Langlade County, WI	Antigo Silt Loam	9.0	Potato/alfalfa rotation	Conventional	16
AO2	Langlade County, WI	Antigo Silt Loam	22.7	Alfalfa/potato/snap bean rotation	Conventional	13
AO3	Langlade County, WI	Antigo Silt Loam	9.1	Potato/snap bean/oats rotation	Conventional	18
MT1	Marathon County, WI	Loyal Silt Loam	3.1	Corn silage/alfalfa rotation	No‐till	36
MT3	Marathon County, WI	Loyal Silt Loam	2.1	Rotationally grazed pasture	None	34
KD1	Kewaunee County, WI	Waymor Silt Loam	1.1	Corn silage/winter wheat/alfalfa	Conventional	9
KD2	Kewaunee County, WI	Waymor Silt Loam	8.0	Corn silage/soybean/ryegrass	Conventional	10
ESW3	Brown County, WI	Waymor Silt Loam	2.6	Alfalfa	None	1
ESW4	Brown County, WI	Oshkosh Silty Clay Loam	2.0	Corn	Conservation	3
ESW5	Brown County, WI	Oshkosh Silty Clay Loam	1.9	Corn	Conservation	5
HSW	Outagamie County, WI	Manawa Silty Clay Loam	8.5	Alfalfa/corn silage	No‐till	3
INSW1	Allen County, IN	Pewamo Silty Clay Loam	1.9	Corn/soybean	No‐till	4
INTL1	Allen County, IN	Pewamo Silty Clay Loam	1.9	Corn/soybean	No‐till	10

*Note*. “No‐till” refers to cropped fields managed without conventional tillage, whereas “none” refers to perennial forage or pasture sites with no tillage operation during the monitoring period.

Abbreviation: WSS, Web Soil Survey.

### 2.2 Data collection and sample processing

Monitoring protocols spanned the full calendar year to capture the distinct hydraulic regimes of the nongrowing season, specifically snowmelt and freeze‐thaw cycling. Adherence to US Geological Survey edge‐of‐field protocols ensured data consistency across the monitoring network (Stuntebeck et al., 2008). Acquisition of runoff samples utilized event‐based triggers to capture a composite sample from storm events. A ChemTrac PC5000 Particle Counter (Environmental Science Research Lab, University of Wisconsin‐Green Bay, WI) was used to quantify particle size through laser obscuration analysis. Prior to analysis, refrigerated samples were pre‐filtered through a 250‐µm sieve to remove large organic debris capable of interfering with optical particle analysis instrumentation. Prevention of sensor saturation and clogging necessitated a 1:99 dilution with ultrapure water. Operational parameters included a flow rate of 75 mL min^−^^1^ with an initial 50 mL purge of deionized water to eliminate cross‐contamination between samples. Measurement of the suspension encompassed 23 distinct size bins ranging from 3 to 126 µm. Final particle concentrations were corrected using a standard dilution equation and primary particle size. Suspended sediment concentration, TP, and dissolved phosphorus (DP) measurements were completed at the University of Wisconsin–Stevens Point Water and Environmental Analysis Lab according to American Society for Testing and Materials standard D‐3977 B, US Environmental Protection Agency method 365.4, and Standard Methods: 4500‐P G, respectively. TP and DP samples were preserved with H_2_SO_4_ and refrigerated until analyzed, and DP samples were filtered through a 0.45‐µm membrane.

Laser diffraction PSDs were converted to mass‑based PSDs because count‑based outputs overweight fine/colloidal particles, whereas settling and BMP performance depend on mass and floc properties. To convert volumes to mass, we did not measure particle/floc density in situ; instead, we used literature‑derived values and reported sensitivity bands. Samples were collected by fixed edge‑of‑field samplers as part of an ongoing multi‐site study; hardware/programming could not be modified to collect the additional volume and paired calibration samples needed for direct density measurement. Quartz mineral density is 2.65 g cm^−^^3^ (U.S. Army Corps of Engineers, 2020); common clays are ∼2.4–2.6 g cm^−^^3^—for example, montmorillonite ≈2.4 g cm^−^^3^ and kaolinite/illite ≈2.6 g cm^−^^3^ (Schwyter & Vaughan, 2021); organic matter is 1.1–1.5 g cm^−^^3^ in surface‑soil runoff matrices (USDA NRCS, 2018); and freshwater flocs commonly contain ∼5%–30% solids, implying *ρ* ≈ 1.05–1.30 g cm^−^^3^ for porous, water‐filled aggregates (Nghiem et al., 2024). For volumetric‑to‑mass conversion, we used *ρ* = 2.0 g cm^−^^3^ as multi‑river intercomparisons that calibrated volumetric (laser) suspended sediment to gravimetric suspended sediment found a best‑fit effective particle density of 2.05 g cm^−^^3^ (Czuba et al., 2023)

### 2.3 Statistical analysis

Distribution (PSD) metrics (D_20_, D_50_, and D_80_) were interpolated from the resulting cumulative percentage curves. Statistical analyses were performed in R 4.3.3 (R Core Team, 2025) and IBM SPSS Statistics 29 (IBM Corp.) to test the influence of management practices and seasonality on particle‐size and water‐quality outcomes. The parent soil served as a fundamental baseline control; by isolating the texture (e.g., Silt Loam and Silty Clay Loam), statistical analysis could attribute observed PSD shifts directly to anthropogenic management decisions rather than geologic heterogeneity. Cropping and tillage regimes functioned as the primary spatial independent variable, ranging from intensively tilled potato and snap bean rotations to conventional row crops and rotationally grazed pasture (Table 1). Inclusion of such diverse systems allowed for a stress test of sediment transport mechanics across a spectrum of soil disturbance intensities. Hydrologic seasonality served as the corresponding temporal variable. Validation of statistical assumptions of normal distribution utilized quantile−quintile plots and Shapiro–Wilk tests.

Failure to meet standard parametric assumptions necessitated a transition to a robust 2500‐iteration bootstrap resampling method to build robust distributions of the means for all three particle metrics (i.e., D_20_, D_50_, and D_80_). A bootstrapped three‐way analysis of variance (ANOVA) model subsequently analyzed the main effects and interactions of Site ID, Season, and Tillage. Evaluation of soil texture percentages utilized the Wilcoxon Rank‐Sum test (Mann–Whitney *U* test) to provide evidence of differences between tillage practices, as these data also failed normality assumptions. Spearman rank correlation identified relationships between site characteristics, such as catchment area and tillage, and water quality parameters. To explicitly rank the relative influence of environmental drivers (e.g., Seasonality vs. Nutrient Yield), a linear mixed‐effects model (LMM) was fitted to the bootstrapped dataset. Standardized coefficients were calculated to allow for direct comparison of effect sizes across variables with differing units.

## 3 RESULTS AND DISCUSSION

### 3.1 Sediment characterization and methodological divergence

**FIGURE 2:**
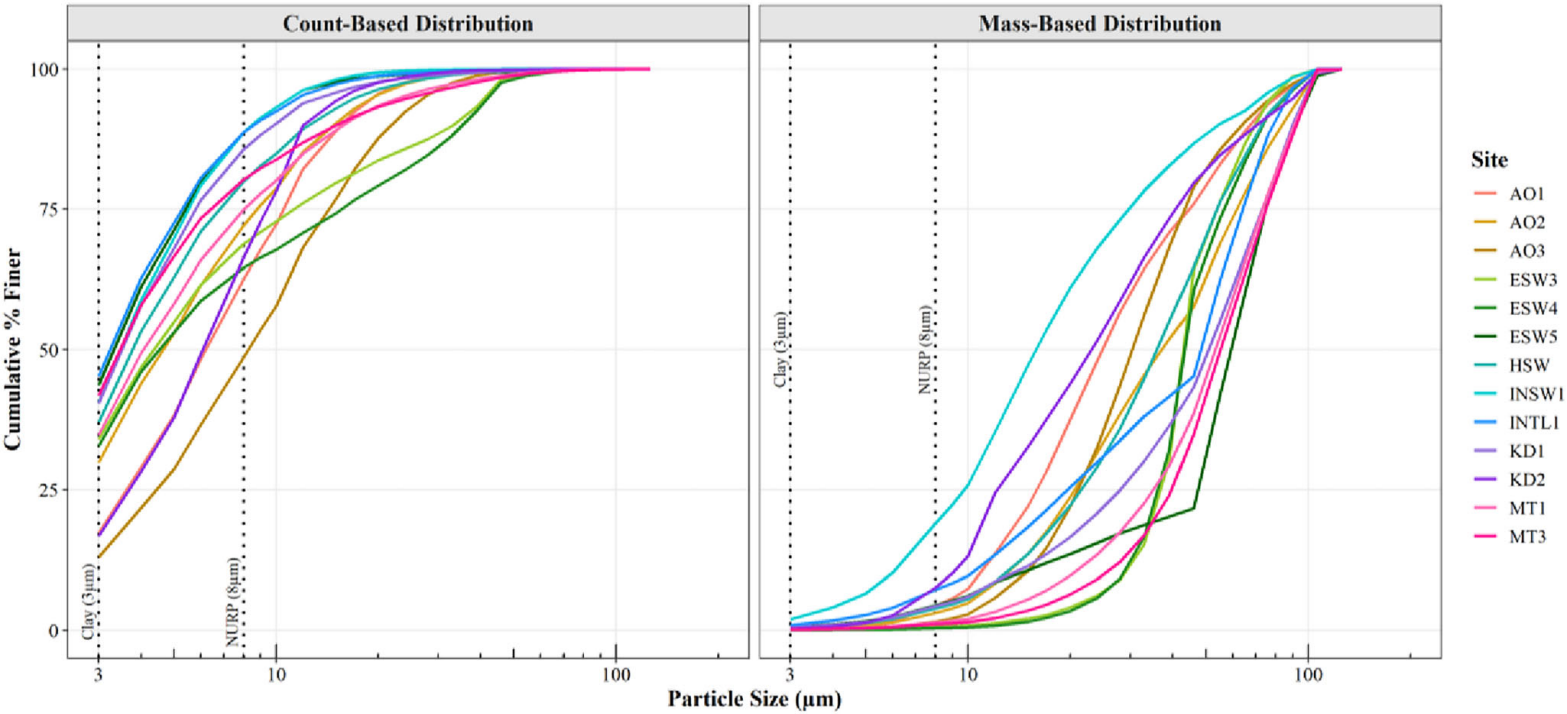
Mass–count discrepancy. Comparison of cumulative particle size distribution (PSD) curves derived from count‐based (left) and mass‐based (right) analysis.

Mass‐ and count‐based PSDs classified the same runoff samples differently (Table 2; Figure 2): volume‐weighted (mass‐based) metrics suggested a coarser influent (mean D_50_ ≈ 30 µm; sand–sandy loam), whereas number‐weighted (count‐based) metrics indicated fine, clay‐rich suspensions (mean D_50_ ≈ 3.5 µm) across sites (Table S1). Volume scales with the cube of particle radius, so a small number of large, low‐density organic aggregates can dominate the mass signal and inflate *D* values, even when the water column is numerically dominated by fine mineral particles (Droppo et al., 2005). Large, low‐density aggregates can occur because suspended sediment often exists as composite flocs made of mineral particles bound with organic detritus and microbial extracellular polymers, creating porous, water‐rich structures that are large in size yet low in effective density (Droppo et al., 1997; Spencer et al., 2021; Walch et al., 2022). In agricultural runoff, these aggregates can originate from eroded soil aggregates that disaggregate and then reflocculate into hybrid aggregate floc particles during transport and from entrained organic debris that contributes to suspended solids (Grangeon et al., 2014). Mass‐based PSDs emphasize the readily settleable fraction, whereas count‐based PSDs better represent the fine fraction most likely to bypass settling; this distinction matters for sizing because required treatment surface area increases as settling velocity decreases $\left(S_{a}=1.2\;q_{o}/v_{s}\right)$, so designs anchored to coarse, mass‐weighted particles can under‐size basins relative to a fine‐particle criterion. As an urban stormwater reference point, Selbig et al. (2016) evaluated the widely accepted Nationwide Urban Runoff Program PSD, with median diameter D_50_ = 8 µm, in stormwater control measure design and showed that PSD assumptions can alter predicted design requirements. Measured runoff PSDs indicate a related but distinct concern: samples can appear relatively coarse on a mass basis while still containing fine, clay‐rich suspensions that are less likely to settle, indicating that parent‐soil‐texture‐based sediment basin assumptions may not fully represent particles transported from working fields.

**TABLE 2:** Parent soil classification and mean D_50_ particle size for runoff collected from each site, comparing mass‐based and count‐based particle size distribution (PSD) interpretations and corresponding runoff textural classifications.

Site ID	WSS parent soil	Mass‐based classification	Count‐based classification	Mass‐based D_50_ (µm)	Count‐based D_50_ (µm)	Mass:count D_50_ ratio
AO1	Antigo Silt Loam	Silt	Silt Loam	24.89	6.24	3.99
A02	Antigo Silt Loam	Sand	Silty Clay Loam	37.20	4.67	7.97
A03	Antigo Silt Loam	Silt	Silt Loam	30.54	8.28	3.69
MT1	Loyal Silt Loam	Sand	Silty Clay Loam	53.44	4.06	13.16
MT3	Loyal Silt Loam	Sandy Loam	Silty Clay	55.21	3.50	15.77
KD1	Waymor Silt Loam	Sand	Silty Clay	51.11	3.55	14.40
KD2	Waymor Silt Loam	Silt	Silt Loam	23.13	6.10	3.79
ESW3	Waymor Silt Loam	Sand	Silty Clay Loam	43.06	4.39	9.81
ESW4	Oshkosh Silty Clay Loam	Sand	Silty Clay Loam	43.38	4.56	9.51
ESW5	Oshkosh Silty Clay Loam	Sandy Loam	Silty Clay	59.46	3.36	17.70
HSW	Manawa Silty Clay Loam	Sand	Silty Clay Loam	36.03	3.80	9.48
INSW1	Pewamo Silty Clay Loam	Silt	Silty Clay	15.87	3.51	4.52
INTL1	Pewamo Silty Clay Loam	Sand	Silty Clay	48.48	3.27	14.83

Abbreviation: WSS, Web Soil Survey.

**FIGURE 3:**
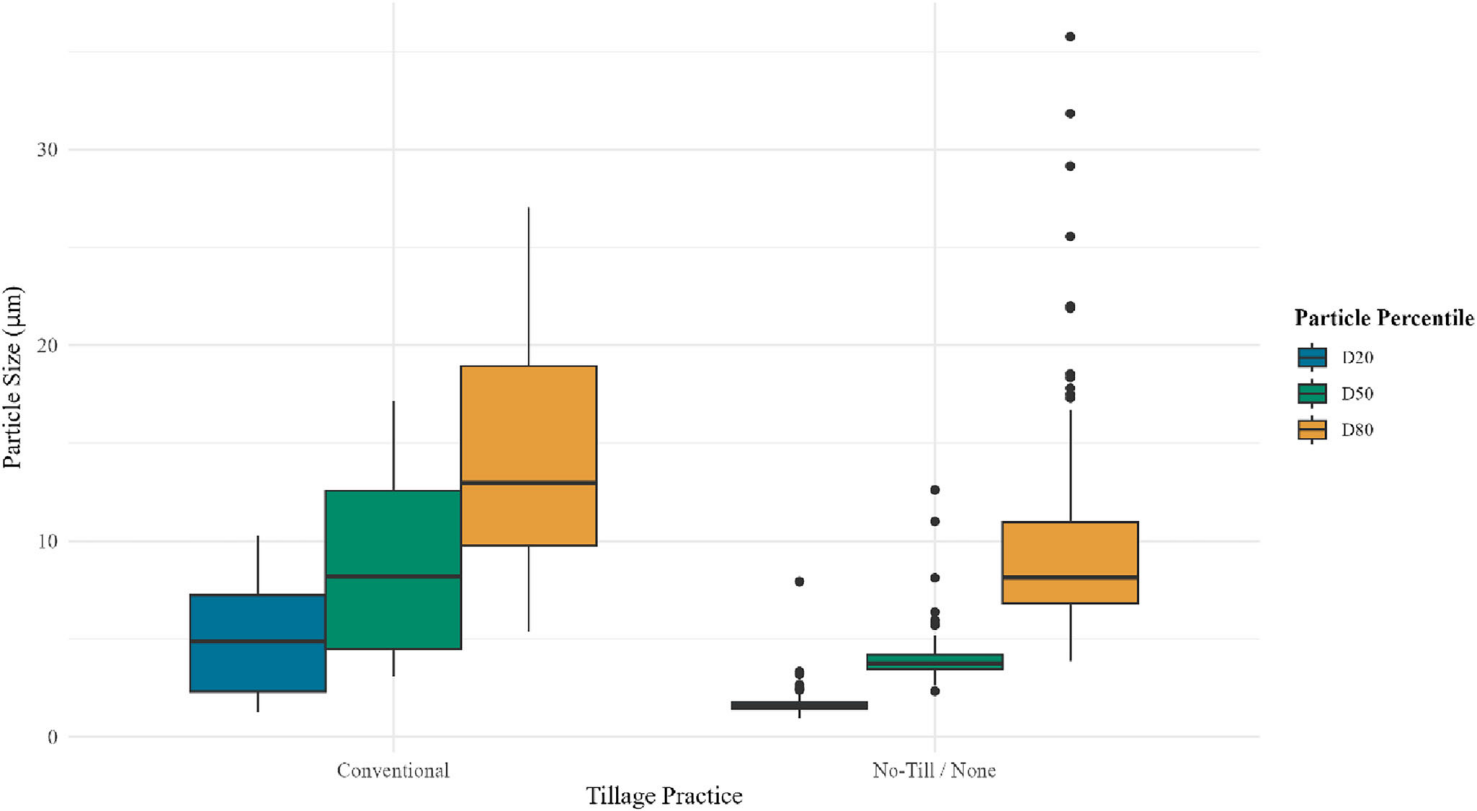
Grouped boxplot of particle size distribution (D_20_, D_50_, and D_80_) aggregated by tillage practice. Conventional tillage (left) exhibits high variance and coarser medians, while no‐till practices (right) show a collapsed interquartile range, indicating the consistent discharge of a fine‐grained, homogeneous clay suspension.

In a previous field study of agricultural runoff treatment in a pond–wetland system, Wisconsin NRCS 350 sedimentation basin sizing for an 80% suspended sediment removal objective in clay soils yielded 44%–72% suspended sediment load reduction, and influent suspended sediment was dominated by very fine particles (D_50_ ≈ 2.8–4 µm), which constrained sedimentation efficiency (Holly et al., 2026). NRCS 350 (Wisconsin) assigns the design settling velocity by dominant soil textural class (sandier, loamy/silty, or clayey); application of this approach produced a modeled plug flow detention time of 2.5 days (Holly et al., 2026). The design standard missed the 80% target because event scale detention was short relative to fine particle settling requirements, with 1–7 µm particles requiring 4.6–19 days to settle to a depth of 0.9 m in the same system (Holly et al., 2026). Wisconsin standards operationalize high sediment removal by targeting fine particles and corresponding settling velocities, including a 3 µm criterion for 80% total suspended solids (TSS) reduction for the previous study design.

Increasing detention beyond 2.5 days would require substantially larger pond volumes (and higher costs) while providing negligible additional treatment benefit for agricultural runoff. Recommended stormwater improvements for agricultural runoff must focus on surface withdrawal plus flocculation because fine particles limit what hydraulics alone can achieve. In controlled basin testing, a skimmer reduced discharged sediment from 26.1 to 14.3 kg (45% reduction) and barriers reduced it to 20.4 kg (22% reduction), yet the effluent PSD matched the influent for particles <75 µm, and retention was poorest in the 6–12 µm range, indicating diminishing returns for fines (Millen et al., 1997). When fines dominate and gravity settling cannot meet TSS or turbidity targets, coagulant dosing is required. Coagulant addition with polyacrylamide (PAM) consistently improved fine sediment treatment, reducing turbidity by 66%–88% and effluent TSS by up to 80% in stilling basin tests, achieving 97.9% event scale sediment removal with the correct PAM formulation in a field‐monitored basin, and reducing turbidity by up to 98% for pumped construction site water (Bhardwaj & McLaughlin, 2008; Fang et al., 2015; Kang et al., 2018).

### 3.2 Hydrologic and agronomic drivers of transport

Particle‐size metrics were non‐normally distributed (*p* < 0.001 for D_20_, D_50_, and D_80_), supporting bootstrapped and nonparametric analyses (Table S2). Soil texture percentages also differed significantly among tillage groups (Table S3). Across events, TP yield decreased with increasing D_50_ (*p* < 0.001) while TN yield increased with D_50_ (*p* = 0.014), indicating that TP and TN were associated with different particle‐size fractions rather than a single shared pattern. In a simulated rainfall study on steep cropland on an entisol, operational fractionation (2‐µm filtration plus ultracentrifugation) indicated that TP can be strongly associated with the colloid size pool (<2 µm); however, it was highly variable (17.2%–83.9% of TP) (He et al., 2019). Colloidal P dominated because TP (and available P) were enriched in the finest (<2 µm) fraction that is readily mobilized and transported in runoff, whereas P associated with coarser particles tended to deposit during transport, reducing its contribution to the exported TP signal (He et al., 2019). TN yield increased with D_50_ because higher D_50_ events likely reflect increased transport of larger, low‐density organic aggregates that are enriched in organic‐associated N, whereas phosphorus remains coupled to persistent fine mineral particles that dominate lower D_50_ events. Fettweis et al. (2022) separated particle organic nitrogen into a labile fraction versus a less reactive mineral‐associated (refractory) fraction and reported that it is the fresh fraction that promotes the formation of larger biomineral flocs. The association of P with fine particles and N with low‐density flocs supports the importance of coagulant modifications of pond–wetland settling. Furthermore, the constructed pond–wetland system treating agricultural runoff showed limited DP removal, with DP load reductions of only 9%–27% (Holly et al., 2026). Coagulant‑enhanced stormwater retrofits have reported approximately 90% TP removal (including DP) and 35%–65% TN removal, indicating greater capture of dissolved nutrient forms than passive settling alone (Herr & Mohandoss, 2023).

In the LMM, seasonality strongly predicted D_50_, with winter events exhibiting significant coarsening relative to spring (*β* = +4.35, 95% confidence interval [2.72, 6.01], *p* < 0.001; Table S4); the observed D_50_ distribution by season is shown in Figure S1, and seasonal patterns across D_20_, D_50_, and D_80_ are shown in Figure S2. Winter coarsening likely reflects snowmelt/runoff over frozen surfaces preferentially mobilizing particulate organic matter and fine particles, which yields 10‐fold higher organic‑matter enrichment during snowmelt than rainfall runoff and can produce larger but low‑density aggregates rather than mineral sand (Droppo, 2001; Panuska & Karthikeyan, 2010). Residue inputs during thaw increase organic material in winter runoff, and these porous, water‐rich aggregates can remain poorly settleable even at larger apparent diameters because settling is strongly influenced by aggregate composition/porosity (Elliott, 2013; Lawrence et al., 2023). Coagulant treatment would improve removal by neutralizing colloids and forming precipitates that bind organic particles into more settleable floc.

Tillage significantly influenced PSDs in the bootstrapped three‐way ANOVA (*p* < 0.001), with conventional tillage producing a heterogeneous mixture (median ∼8–9 µm; interquartile range extending to ∼20 µm) while no‐till produced fine suspension (D_50_ ≈ 4 µm) with less variability (Figure 3) (Table S5). Soil texture percentages differed by tillage practice, suggesting that parent‐soil texture may partly contribute to observed PSD differences among tillage groups. Mechanistically, this fining is consistent with selective retention of coarse aggregates under no‐till (due to improved near‐surface soil structure infiltration), leaving the fine mineral fraction as the dominant exported material (Blanco‐Canqui & Ruis, 2018). A complementary process is raindrop‐driven aggregate breakdown, which can enrich exported sediment in fines (e.g., eroded sediment >50% fines even when source soil is <5% fines) (Ghadiri & Rose, 1991). Prior field rainfall simulations similarly found significantly lower sediment under no‐till than under chisel/disk tillage, consistent with reduced mobilization of coarse fractions and a finer residual suspension when material is exported (Verbree et al., 2010). Given that no‐till can reduce rainfall‐driven sediment delivery by ∼93% (0.24 vs. 3.46 t ha^−^^1^ h^−^^1^ under simulated heavy rainfall) (Seitz et al., 2019), the mass available for capture in a pond–wetland system can become small enough that cost‐effectiveness declines sharply. Using the Holly et al. (2026) cost framework, conservation tillage is $0.37 kg^−^^1^ TSS removed, and a pond–wetland system is $0.15 kg^−^^1^ TSS removed; however, because no‐till can reduce sediment delivery by ∼93%, the marginal cost effectiveness of a pond–wetland system on a low‐erosion no‐till field increases to ∼$2.2 kg^−^^1^ TSS removed (exceeding the per kg TSS costs of the other BMP compared in the study), indicating pond–wetland systems are most justified for conventionally tilled row‐crop watersheds.

## 4 CONCLUSION

Agricultural runoff PSDs across 13 Upper US Midwest edge‐of‐field watersheds showed a consistent mass–count mismatch: volume‐weighted metrics suggested relatively coarse influent, while number‐weighted metrics indicated clay‐rich, colloid‐dominated suspensions. The observed mass–count divergence indicates that generalized stormwater PSD benchmarks and parent‐soil‐texture‐based settling assumptions may not fully represent agricultural runoff particles. Fine‐particle dominance helps explain limits of sedimentation‐focused treatment and supports a treatment train approach that targets both settleable aggregates and persistent colloids. Seasonality significantly predicted D_50_, with winter events showing coarsening relative to the baseline season. Winter coarsening likely reflects mobilization of low‐density organic flocs that increase apparent particle size without proportional settling benefit. Nutrient partitioning also differed by fraction, with phosphorus tracking fine mineral particles and nitrogen aligning with larger organic aggregates. Laser obscuration reduces aggregation‐breaking bias relative to pipette methods but assumes spherical geometry for equivalent spherical diameters, which limits interpretation. Future work should apply wet sieving and filtration‐based fractionation to quantify mass‐based size fractions and particle‐associated nutrients by size class, measure effective aggregate density, and test treatment‐train performance in basins with coagulation addition.

## AUTHOR CONTRIBUTIONS

**Michael A. Holly**: Conceptualization; formal analysis; funding acquisition; investigation; methodology; project administration; resources; supervision; validation; visualization; writing—original draft; writing—review and editing. **Andrew J. Votis**: Data curation; formal analysis; writing—original draft. **Mason Anderson‐Giles**: Data curation; software. **Kpoti M. Gunn**: Formal analysis; supervision; validation.

## CONFLICT OF INTEREST STATEMENT

The authors declare no conflicts of interest.

## Supporting information



Supplemental material includes Tables S1–S5 and Figures S1–S2. Tables S1–S5 report particle count percentages by size bin (Table S1), distributional testing (Table S2), soil texture comparisons by tillage (Table S3), and model outputs from the linear mixed‐effects model and bootstrapped three‐way ANOVA (Tables S4–S5). Figures S1–S2 summarize seasonal D_50_ distributions and seasonal D_20_, D_50_, D_80_ distributions, respectively.
