# Supplementary material for: Characterizing particle size in agricultural runoff to inform stormwater basin design
Source: J Environ Qual. 2026 Sep 21;55(5):e70267. doi: 10.1002/jeq2.70267 (PMC13594369; doi:10.1002/jeq2.70267)
Supplement: Supplementary file 1 [file JEQ2-55-0-s001.docx]

**Supplemental materials**

*Table S1. Descriptive statistics for particle count percentages across eight size bins (µm) for 13 monitoring sites.*

| **Site ID** | Statistic | >125 µm | 65-125 µm | 33-65 µm | 15-33 µm | 8-15 µm | 5-8 µm | 3-5 µm | < 3 µm | Total Count |
| --- | --- | --- | --- | --- | --- | --- | --- | --- | --- | --- |
| **AO1 (Antigo)** | No. of Obs | 16 | 16 | 16 | 16 | 16 | 16 | 16 | 16 | 16 |
|  | Minimum | 0 | 0.01 | 0.03 | 0.74 | 7.81 | 9.67 | 3.37 | 0.85 | 20,930 |
|  | Maximum | 0 | 3.43 | 9.08 | 47.22 | 45.06 | 30.95 | 29.87 | 48.17 | 2,819,438 |
|  | Median | 0 | 0.26 | 1.97 | 6.78 | 12.53 | 14.61 | 22.67 | 37.45 | 72,423 |
|  | Mean | 0 | 0.51 | 3.03 | 12.52 | 16.77 | 15.83 | 20.17 | 31.18 | 558,026 |
|  | St. Dev. | 0 | 0.84 | 3.07 | 14.08 | 10.62 | 5.5 | 7.75 | 16.25 | 1,026,775 |
| **AO2 (Antigo)** | No. of Obs | 13 | 13 | 13 | 13 | 13 | 13 | 13 | 13 | 13 |
|  | Minimum | 0 | 0.01 | 0.31 | 4.47 | 9.84 | 12.27 | 5.65 | 1.74 | 20,870 |
|  | Maximum | 0 | 0.93 | 7.09 | 41 | 41.69 | 25.21 | 26.88 | 44.63 | 1,344,770 |
|  | Median | 0 | 0.13 | 1.19 | 6.12 | 14.62 | 16.89 | 24.42 | 34.84 | 211,786 |
|  | Mean | 0 | 0.25 | 1.97 | 11.86 | 18.17 | 17.39 | 21.1 | 29.25 | 271,954 |
|  | St. Dev. | 0 | 0.3 | 2.03 | 11.88 | 10.49 | 3.22 | 6.95 | 14.23 | 337,996 |
| **AO3 (Antigo)** | No. of Obs | 18 | 18 | 18 | 18 | 18 | 18 | 18 | 18 | 18 |
|  | Minimum | 0 | 0 | 0.06 | 0.18 | 8.4 | 8.74 | 2.76 | 0.66 | 19,217 |
|  | Maximum | 0 | 0.59 | 9.56 | 49.48 | 43.58 | 28.77 | 32.05 | 46.26 | 3,001,709 |
|  | Median | 0 | 0.04 | 0.68 | 11.06 | 27.54 | 20.23 | 18.7 | 12.75 | 2,412,479 |
|  | Mean | 0 | 0.08 | 1.94 | 17.76 | 25.93 | 19.75 | 17.4 | 17.13 | 1,834,740 |
|  | St. Dev. | 0 | 0.14 | 2.59 | 16.81 | 11.68 | 6.35 | 10.36 | 16.23 | 1,082,830 |
| **ESW3 (East River)** | No. of Obs | 1 | 1 | 1 | 1 | 1 | 1 | 1 | 1 | 1 |
|  | Minimum | 0 | 0.33 | 9.74 | 10.21 | 10.9 | 13.87 | 20.91 | 34.04 | 44,681 |
|  | Maximum | 0 | 0.33 | 9.74 | 10.21 | 10.9 | 13.87 | 20.91 | 34.04 | 44,681 |
|  | Median | 0 | 0.33 | 9.74 | 10.21 | 10.9 | 13.87 | 20.91 | 34.04 | 44,681 |
|  | Mean | 0 | 0.33 | 9.74 | 10.21 | 10.9 | 13.87 | 20.91 | 34.04 | 44,681 |
|  | St. Dev. | NA | NA | NA | NA | NA | NA | NA | NA | NA |
| **ESW4(East River)** | No. of Obs | 3 | 3 | 3 | 3 | 3 | 3 | 3 | 3 | 3 |
|  | Minimum | 0 | 0.01 | 0.04 | 1.28 | 9.48 | 11.23 | 20.32 | 22.59 | 41,293 |
|  | Maximum | 0 | 0.78 | 14.25 | 15.41 | 16.08 | 29.52 | 30.48 | 33.86 | 2,525,370 |
|  | Median | 0 | 0.2 | 8.65 | 12.28 | 9.86 | 11.7 | 20.36 | 31.63 | 44,612 |
|  | Mean | 0 | 0.33 | 7.65 | 9.66 | 11.8 | 17.48 | 23.72 | 29.36 | 870,425 |
|  | St. Dev. | 0 | 0.4 | 7.16 | 7.42 | 3.71 | 10.43 | 5.85 | 5.96 | 1,433,225 |
| **ESW5(East River)** | No. of Obs | 5 | 5 | 5 | 5 | 5 | 5 | 5 | 5 | 5 |
|  | Minimum | 0 | 0.01 | 0.07 | 1.15 | 7.22 | 15.15 | 23.46 | 17.71 | 28,248 |
|  | Maximum | 0 | 0.79 | 3.25 | 5.55 | 22.14 | 30.21 | 28.68 | 46.28 | 779,241 |
|  | Median | 0 | 0.38 | 1.69 | 2.79 | 11.27 | 16.58 | 24.85 | 41.63 | 132,600 |
|  | Mean | 0 | 0.43 | 1.79 | 3.35 | 12.6 | 19 | 25.33 | 37.49 | 236,171 |
|  | St. Dev. | 0 | 0.33 | 1.35 | 1.71 | 5.69 | 6.3 | 1.96 | 11.44 | 308,081 |
| **HSW (Outagamie)** | No. of Obs | 3 | 3 | 3 | 3 | 3 | 3 | 3 | 3 | 3 |
|  | Minimum | 0 | 0.01 | 0.33 | 5.14 | 10.9 | 13.39 | 21.91 | 35.18 | 30,075 |
|  | Maximum | 0 | 0.4 | 5.01 | 12.8 | 13.46 | 17.58 | 26.44 | 39.87 | 349,237 |
|  | Median | 0 | 0.3 | 2.11 | 6.65 | 11.42 | 15.34 | 24.73 | 37.06 | 42,367 |
|  | Mean | 0 | 0.23 | 2.48 | 8.19 | 11.93 | 15.44 | 24.36 | 37.37 | 140,560 |
|  | St. Dev. | 0 | 0.2 | 2.36 | 4.06 | 1.35 | 2.09 | 2.29 | 2.36 | 180,824 |
| **INSW1 (Indiana)** | No. of Obs | 4 | 4 | 4 | 4 | 4 | 4 | 4 | 4 | 4 |
|  | Minimum | 0 | 0 | 0.03 | 1.19 | 6.63 | 15.62 | 23.82 | 34.36 | 90,549 |
|  | Maximum | 0 | 0.13 | 1.15 | 8.18 | 15.12 | 20.02 | 30.24 | 47.92 | 1,383,004 |
|  | Median | 0 | 0 | 0.05 | 1.72 | 10.17 | 17.6 | 28.06 | 40.21 | 360,802 |
|  | Mean | 0 | 0.03 | 0.32 | 3.2 | 10.52 | 17.71 | 27.54 | 40.67 | 548,789 |
|  | St. Dev. | 0 | 0.07 | 0.56 | 3.34 | 3.52 | 1.83 | 2.73 | 5.7 | 571,080 |
| **INTL1 (Indiana)** | No. of Obs | 10 | 10 | 10 | 10 | 10 | 10 | 10 | 10 | 10 |
|  | Minimum | 0 | 0 | 0.01 | 0.19 | 3.95 | 12.72 | 22.6 | 39.06 | 11,467 |
|  | Maximum | 0 | 0.69 | 3.2 | 9.84 | 12.56 | 18.19 | 28.79 | 54.76 | 475,095 |
|  | Median | 0 | 0.03 | 0.2 | 2.45 | 9.65 | 16.11 | 26.92 | 43.92 | 132,764 |
|  | Mean | 0 | 0.09 | 0.56 | 3.02 | 8.86 | 15.65 | 26.66 | 45.17 | 176,735 |
|  | St. Dev. | 0 | 0.21 | 0.97 | 2.88 | 3.1 | 1.79 | 1.8 | 5.32 | 145,288 |
| **KD1 (Kewaunee)** | No. of Obs | 9 | 9 | 9 | 9 | 9 | 9 | 9 | 9 | 9 |
|  | Minimum | 0 | 0.01 | 0.19 | 1.32 | 8.82 | 12.19 | 22.05 | 34.43 | 11,566 |
|  | Maximum | 0 | 1.78 | 9.5 | 9.35 | 14.05 | 19.01 | 28.79 | 45.21 | 791,182 |
|  | Median | 0 | 0.07 | 0.83 | 4.06 | 10.28 | 16.56 | 26.33 | 39.94 | 82,867 |
|  | Mean | 0 | 0.47 | 2.17 | 5.05 | 10.7 | 16.4 | 25.83 | 39.38 | 235,036 |
|  | St. Dev. | 0 | 0.64 | 3.08 | 2.87 | 1.61 | 2.02 | 2.6 | 3.5 | 264,378 |
| **KD2 (Kewaunee)** | No. of Obs | 10 | 10 | 10 | 10 | 10 | 10 | 10 | 10 | 10 |
|  | Minimum | 0 | 0 | 0.03 | 0.99 | 4.65 | 11.31 | 13.28 | 5.03 | 40,595 |
|  | Maximum | 0 | 1.1 | 7.21 | 12.71 | 44.08 | 33.69 | 29.12 | 55.98 | 2,761,645 |
|  | Median | 0 | 0.04 | 0.42 | 5.32 | 12.23 | 15.14 | 24.55 | 36.3 | 235,747 |
|  | Mean | 0 | 0.18 | 1.51 | 5.45 | 15.18 | 18.15 | 23.81 | 35.73 | 765,419 |
|  | St. Dev. | 0 | 0.35 | 2.44 | 4.01 | 11.68 | 7.55 | 4.71 | 16.25 | 1,032,942 |
| **MT1 (Marathon)** | No. of Obs | 36 | 36 | 36 | 36 | 36 | 36 | 36 | 36 | 36 |
|  | Minimum | 0 | 0 | 0.08 | 0.73 | 5.04 | 8.99 | 15.74 | 25.09 | 2,971 |
|  | Maximum | 0 | 4.5 | 16.45 | 20.26 | 19.38 | 19.52 | 31.05 | 56.96 | 850,379 |
|  | Median | 0 | 0.23 | 1.98 | 7.66 | 12.22 | 14.8 | 23.21 | 36.05 | 81,109 |
|  | Mean | 0 | 0.73 | 3.75 | 8.61 | 12.47 | 15.06 | 22.75 | 36.63 | 124,269 |
|  | St. Dev. | 0 | 0.99 | 4.08 | 4.23 | 3.52 | 2.99 | 3.15 | 6.68 | 156,763 |
| **MT3 (Marathon)** | No. of Obs | 34 | 34 | 34 | 34 | 34 | 34 | 34 | 34 | 34 |
|  | Minimum | 0 | 0 | 0.18 | 0.81 | 1.85 | 6.19 | 14.09 | 24.67 | 6,536 |
|  | Maximum | 0 | 3.1 | 19.3 | 22.2 | 20.68 | 20.98 | 31.72 | 64.42 | 278,237 |
|  | Median | 0 | 0.27 | 1.84 | 5.89 | 10.29 | 14.57 | 24.31 | 39.9 | 45,523 |
|  | Mean | 0 | 0.54 | 3.6 | 7.7 | 10.58 | 14.27 | 23.77 | 39.54 | 65,460 |
|  | St. Dev. | 0 | 0.73 | 4.31 | 5.15 | 3.58 | 3.22 | 3.63 | 7.17 | 58,827 |

*Table S2. Shapiro–Wilk normality test results for residuals from the standard ANOVA model for D20, D50, and D80.*

| Metric (um) | Statistic | P Value |
| --- | --- | --- |
| D_20_ | 0.72997 | < 0.001 |
| D_50_ | 0.797841 | < 0.001 |
| D_80_ | 0.907831 | < 0.001 |

*Table S3. Wilcoxon rank‑sum test (Mann–Whitney U) results for soil texture percentages by tillage practice.*

| **Metric** | **W-Statistic** | **p-value** | **Significant?** |
| --- | --- | --- | --- |
| Clay Percentage | 520 | < 0.001 | Yes |
| Silt Percentage | 3000 | < 0.001 | Yes |
| Sand Percentage | 1352 | 0.058 | Marginally |

*Table S4. Linear mixed‑effects model results predicting D50; estimates are standardized coefficients with 95% bootstrap confidence intervals (n = 2,500).*

| Predictor | Estimate (β) | 95% CI | P-Value | Significance |
| --- | --- | --- | --- | --- |
| Seasonality |  |  |  |  |
| Winter (vs. Spring) | +4.35 | [2.72, 6.01] | < 0.001 | *** |
| Summer (vs. Spring) | -0.81 | [-1.83, 0.19] | 0.118 | ns |
| Water Chemistry |  |  |  |  |
| Total Phosphorus (TP) | -10.96 | [-15.54, -6.64] | < 0.001 | *** |
| Total Nitrogen (TN) | +2.36 | [0.93, 3.86] | 0.014 | * |
| TSS Yield | +2.76 | [0.78, 4.71] | 0.012 | * |
| Management |  |  |  |  |
| Manure Application | -0.88 | [-1.79, 0.04] | 0.062 | ns |
| Tillage (No-Till) | -2.63 | [-5.50, 0.13] | 0.082 | ns |

*Table S5. Bootstrapped three‑way ANOVA results for D20, D50, and D80, including main effects and interactions for Site ID, Season, and Tillage.*

| Metric | Factor | Sum Sq | Df | F value | p-value |
| --- | --- | --- | --- | --- | --- |
| D_20_ (Target Particle) | Site ID | 13,010 | 12 | 2,377 | < 0.001 |
|  | Season | 16,161 | 3 | 11,810 | < 0.001 |
|  | Tillage | 54,679 | 1 | 119,873 | < 0.001 |
|  | Site ID : Season | 9,982 | 15 | 1,459 | < 0.001 |
|  | Site ID : Tillage | 2,654 | 2 | 2,909 | < 0.001 |
|  | Season : Tillage | 2,239 | 1 | 4,909 | < 0.001 |
|  | Site ID : Season : Tillage | 1,139 | 2 | 1,248 | < 0.001 |
| D_50_ (Median Particle) | Site ID | 25,433 | 12 | 1,925 | < 0.001 |
|  | Season | 46,334 | 3 | 14,027 | < 0.001 |
|  | Tillage | 139,599 | 1 | 126,787 | < 0.001 |
|  | Site ID : Season | 29,704 | 15 | 1,799 | < 0.001 |
|  | Site ID : Tillage | 3,910 | 2 | 1,776 | < 0.001 |
|  | Season : Tillage | 9,024 | 1 | 8,196 | < 0.001 |
|  | Site ID : Season : Tillage | 4,554 | 2 | 2,068 | < 0.001 |
| D_80_ (Larger Particle) | Site ID | 198,253 | 12 | 2,427 | < 0.001 |
|  | Season | 183,509 | 3 | 8,987 | < 0.001 |
|  | Tillage | 255,160 | 1 | 37,487 | < 0.001 |
|  | Site ID : Season | 139,444 | 15 | 1,366 | < 0.001 |
|  | Site ID : Tillage | 2,982 | 2 | 219 | < 0.001 |
|  | Season : Tillage | 20,357 | 1 | 2,991 | < 0.001 |
|  | Site ID : Season : Tillage | 11,182 | 2 | 821 | < 0.001 |

*Figure S1. Observed D50 distribution by season (Spring, Summer, Winter).*

*Figure S2. Seasonal distributions of D20, D50, and D80 (boxplots) aggregated by season.*
